# Authentication and Quality Control of the Brazilian Traditional Herb ‘Carquejas’ (*Baccharis* Species) Using Morpho-Anatomy and Microscopy

**DOI:** 10.3390/plants13213030

**Published:** 2024-10-30

**Authors:** Kevin Alves Antunes, Vijayasankar Raman, Wilmer Hervet Perera, Gustavo Heiden, Roberto Pontarolo, Paulo Vitor Farago, Ikhlas Ahmed Khan, Jane Manfron

**Affiliations:** 1 Postgraduate Program in Pharmaceutical Sciences, State University of Ponta Grossa, 4748 Carlos Cavalcanti Avenue, Ponta Grossa 84030-900, PR, Brazil; kevinalvesantunes@gmail.com (K.A.A.); pvfarago@gmail.com (P.V.F.); 2 National Identification Services, U.S. Department of Agriculture, Washington, DC 20560, USA; vijay.raman@usda.gov; 3 CAMAG Scientific, Inc., 515 Cornelius Harnett Dr, Wilmington, NC 28401, USA; wilmer.perera@camag.com; 4 Embrapa Clima Temperado, BR-392, km 78, Pelotas 96001-970, RS, Brazil; gustavo.heiden@embrapa.br; 5 Department of Pharmacy, Universidade Federal do Paraná, 623, Prefeito Lothário Meissner Avenue, Curitiba 80210-170, PR, Brazil; pontarolo@ufpr.br; 6 National Center for Natural Products Research, School of Pharmacy, University of Mississippi, University, MS 38677, USA; ikhan@olemiss.edu

**Keywords:** anatomy, Asteraceae, *Baccharis*, carqueja, micromorphology, quality control

## Abstract

This research investigates the morpho-anatomical characteristics of seven *Baccharis* species, namely *B. articulata*, *B. milleflora*, *B. myriocephala*, *B. pentaptera*, *B. riograndensis*, *B. sagittalis* and *B. trimera*. Commonly called carquejas, these species have aerial photosynthetic winged stems known as cladodes and are widely used traditionally to treat digestive and diuretic disorders. This study aimed to characterize these commonly misidentified species using morphological and microscopic techniques, including light and scanning electron microscopy coupled with energy-dispersive X-ray spectroscopy. Trichomes, the presence or absence of oil bodies, and a subepidermal collenchyma layer at the wing edge were identified as primary anatomical markers that can help differentiate the studied species.

## 1. Introduction

One of the ten most representative genera of the Asteraceae family is *Baccharis* L., with about 442 accepted species of trees, shrubs, lianas and subshrubs [1]. In Brazil, 180 species are found in the Caatinga, Cerrado, Amazon Forest, Atlantic Forest, Pampa, and Pantanal biomes, being well distributed in all Brazilian regions [2].

Recently, the genus *Baccharis* was divided into seven subgenera and 47 sections, based on a phylogenetic reconstruction. Carquejas belong to two genetically distinct, although morphologically convergent, lineages classified in two sections: *Baccharis* sect. *Caulopterae* (15 species) and *B.* sect. *Aphyllae* (35 species) [3]. The authors stated the need for morphoanatomical studies to differentiate and characterize the two sections, especially those taxa with winged stems, as they are morphologically similar, even though evolutionarily distinct.

Many species of *Baccharis* with regular stems and leaves are known as “vassouras”; while others with modified aerial photosynthetic stems or cladodes are called “carquejas”. Due to the similar morphology and the use of the same common name for different species in each group, they are often mistakenly used in traditional medicine for the same therapeutic purposes [4,5].

Confusion arising from the use of the same common name and the morphological similarity of plants facilitates the misidentification of medicinal plants, which is concerning because many species lack pharmacological and toxicological studies to ensure their therapeutic efficacy and safety. Consequently, the misidentification of a plant species used for medicinal purposes poses a public health risk. Misidentification can occur in two ways, either intentionally as an adulteration, which occurs when profit is sought, for example, by adding different plant parts of the same species (such as roots when the medicinal use refers to the aerial parts), or by incorporating parts from other species, which may not have the same medicinal value. Other plants are erroneously collected through extractivism due to the collector’s lack of necessary knowledge for accurate species identification [5].

Carquejas are aromatic plants that produce essential oils and are used in traditional medicine as a digestive and diuretic [5]. *Baccharis trimera* (Less.) DC., followed by *B. articulata* (Lam.) Pers., are the widely marketed medicinal plants in Brazil, while other species are commonly used locally, such as *B. crispa* Spreng. in Uruguay and *B. genistelloides* (Lam). Pers. along the Andean countries [6,7]. In addition, the adulteration or substitution of this plant material with *B. articulata* (syn. *B. gaudichaudiana* DC.), *B. microcephala* (Less.) DC., and *B. crispa* [8,9] have also been reported in Argentina. Only three species have had their morpho-anatomical and chemical–pharmacopeial parameters studied for quality control, namely *B. crispa* and *B. articulata*, which are found in the Argentine Pharmacopoeia, and *B. trimera*, listed in the Brazilian Pharmacopoeia. *Baccharis trimera* is also included in the National List of Medicinal Plants of Interest to the Unified Health System (RENISUS) [10] and Formulário de Fitoterápicos da Farmacopeia Brasileira (Pharmacotherapeutic Formulary of the Brazilian Pharmacopeia) [11].

In general, species of carquejas exhibit cladodes with 2 to 5 wings along the stem axis. For instance, *B. articulata* typically presents 2 wings, *B. sagittalis* (Less.) DC. has 3, and *B. pentaptera* (Less.) DC. may exhibit 2, 3, or 5 wings. These winged stems are generally leafless or possess sparse leaves reduced to scales, as observed in *B. junciformis* DC. Anatomical characteristics play a crucial role in distinguishing carquejas, including epidermal features such as the contour of anticlinal epidermal cell walls, stomatal types, trichome morphology, mesophyll organization, and the presence of various crystal morphotypes [5].

Several anatomical studies have contributed to the taxonomic differentiation of carqueja species. Budel et al. [12], conducted a comprehensive review of the anatomy of *Baccharis* species, while Freire [13] examined epidermal characteristics in 33 species, including four carquejas: *B. articulata*, *B. crispa*, *B. microcephala* and *B. trimera*. Petenatti et al. [14] provided anatomical distinctions between *B. sagittalis* and *B. triangularis*, highlighting the stomatal index. Subsequently, Rodrigues, Gattuso and Gattuso [15] compared *B. crispa* and *B. trimera*, determining quantitative new micrographic characteristics, and these authors later expanded their analysis to include *B. articulata* and *B. trimera* using morpho-anatomical parameters, the polypeptide profile, and spectrophotometric data. Budel and Duarte [16] contributed further by differentiating *B. microcephala* and *B. trimera* by their morphology features and type of non-glandular trichomes. In 2010, Budel et al. [17] provided macro and microscopic characters of the aerial vegetative organs of *B. junciformis* (syn. *B. usterii* Heering). Additionally, Rodrigues, Gattuso and Gattuso [18] studied the *Baccharis* section *Caulopterae*, producing micrographs of *B. articulata*, *B. crispa*, *B. microcephala*, and *B. trimera* to establish reference standards for quality control of raw materials. In 2018, a morpho-anatomical and statistical analysis of the following *Baccharis* species from sect. *Caulopterae* was conducted: *B. articulata*, *B. crispa*, *B. microcephala*, *B. penningtonii*, *B. phyteumoides*, *B. sagittalis* (Less.) DC., *B. triangularis* and *B. trimera* [19]. Other carquejas such as *B. glaziovii* [20], *B. milleflora* (Less.) DC. [21], *B. trimera* [22], as well as *B. aracatubaensis*, *B. burchellii*, and *B. organensis* [23], have also been subjected to morpho-anatomical studies. A recent book chapter [5] provides a comprehensive review of the morpho-anatomical characteristics of the *Baccharis* species and highlights the need for further comparative studies to enhance understanding of species diversity and improve the taxonomic classification of the genus.

Despite the substantial body of research on the morpho-anatomy of carquejas, challenges remain in the accurate identification of raw material. The high number of carqueja species in Brazil, coupled with their morphological similarity and the use of the same common name for different species, frequently leads to errors during collection, resulting in misidentification and the unintended adulteration of raw materials [5].

Carquejas rank among the most extensively commercialized medicinal plants in Brazil. Considering the morphological similarities and confusing folk nomenclature of the carqueja species, this study aimed to analyze the morphological, microscopic, and histochemical characteristics of seven Brazilian carqueja species, namely *B. articulata*, *B. milleflora*, *B. myriocephala* DC., *B. pentaptera*, *B. riograndensis* Malag. and J.Vidal, *B. sagittalis*, and *B. trimera*, using light and scanning electron microscopy. This study provides pharmacobotanical data on cladodes to support the authentication and quality control of plant raw materials and commercial products containing carquejas.

## 2. Materials and Methods

### 2.1. Plant Material

Fresh samples from seven *Baccharis* species were collected from various geographical regions in Brazil, and the herbarium specimens (Table 1) were identified by taxonomist Dr. Gustavo Heiden. Access to the botanical materials was approved and licensed by CGEN/SISGEN and registered under the code A8364D1.

### 2.2. Preparation of Samples for Light Microscopy (LM)

Fresh cladodes were collected (five specimens from each species) and fixed in formalin, acetic acid, 70% ethanol, 5:5:90 *v*/*v*/*v* (FAA 70) for 3 days [24] and stored in 70% ethanol solution (*v*/*v*) [25]. The samples were washed in distilled water, and at least ten transverse sections were prepared free-hand using razors. The sections were double-stained with Astra blue and basic fuchsin [26], or only with toluidine blue [27]. Then, the sections were mounted on glass slides in a drop of 50% glycerin [28], covered with a coverslip, and sealed with transparent nail polish.

To analyze epidermal surfaces, minor sections of the cladodes were washed and cleared in a 50% hypochlorite solution until translucent. The cladodes were washed again with distilled water and neutralized in an acetic acid solution (5%). The sections were rewashed with distilled water (6×), stained in safranin [28] and mounted as described above.

Birefringent elements, like calcium oxalate crystals, were observed using a polarized light microscope (Nikon E600 POL) equipped with Nikon DSFiv camera systems and Nikon imaging software NIS-Elements AR 4.30 (Nikon Inc., Tokyo, Japan).

### 2.3. Micro-Measurements

The stomatal index (SI) was calculated following the procedure outlined in the Brazilian Pharmacopoeia [29]. Fifteen fields for each species (cladodes) were examined using the formula SI = (S/(S + E)) × 100, where S represents the number of stomata per unit area, and E represents the number of epidermal cells in the same unit area (1 mm^2^). The measurements were performed at a 40× magnification. The same was used for tufts of trichomes. To quantify the mean and standard deviation of stomatal size, all stomata from the ten sampled fields were systematically measured for length and width on the cladodes of each specimen using ImageJ^®^ 1.53t software. A one-way ANOVA, followed by post hoc Tukey’s HSD test, was used to evaluate the presence of statistically significant differences among species, with a significance established at *p* < 0.05.

### 2.4. Histochemical Analysis

Secondary metabolites were investigated by histochemical methods. Photomicrographs were captured using an Olympus CX 31 light microscope with a C-7070 control unit (Olympus Corporation, Tokyo, Japan). Ten transverse sections of fresh cladodes for each species were used for histochemical analysis. The following reagents and stains were used in the histochemical tests: ferric chloride [25] and potassium dichromate [30] to detect phenolic compounds; phloroglucinol/HCl to show lignin [31]; Sudan III [32], Sudan Black [33], Nile blue [34] and Oil red [35] for lipophilic components; iodine solution to detect starch [25]; ruthenium red for pectins [24]; Coomassie brilliant blue [36] and Xylidine Ponceau [37] for protein bodies; Vanillin for condensed tannin [38]; Dragendorff [39], Ellram and Wagner [40] for alkaloids; PAS (periodic acid-Schiff) [41] for polysaccharide; and NADI [42] for terpenes.

### 2.5. Scanning Electron Microscopy (SEM) and Energy Dispersive X-Ray Spectroscopy (EDS)

The FAA-fixed samples were dehydrated by passing through increasing concentrations of (60, 70, 80, 90, and 100%) ethanol in water and dried in a Balzers CPD 030 critical point dryer (BAL-TEC AG, Balzers, Liechtenstein) supplied with liquid CO_2_. The fully dried samples were then coated with gold using a Quorum (model SC7620) sputter coater (Quorum Technologies, Laughton, UK). The specimens were analyzed and photomicrographs were recorded using a Mira 3 Field-Emission SEM (Tescan, Brno-Kohoutovice, Czech Republic) at an accelerating voltage of 15 keV. A chemical analysis of the crystals was performed using an EDS attached to the SEM with the system of Electron Backscatter Diffraction (EBSD).

## 3. Results and Discussion

### 3.1. Analysis of Cladodes

**Figure 1:**
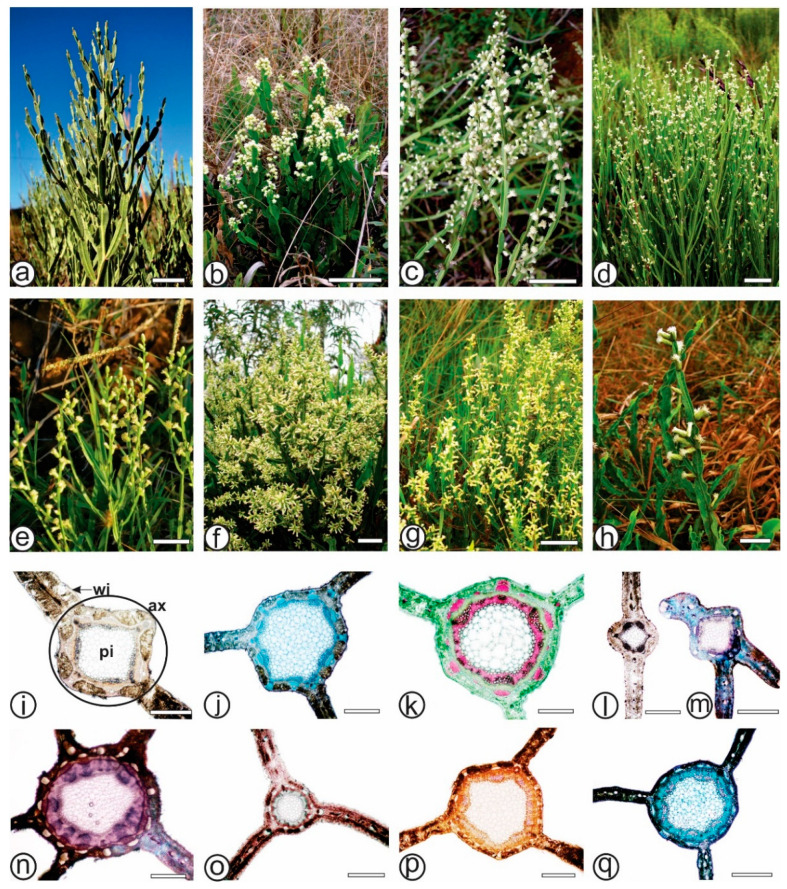
Morpho-anatomy of cladodes of *Baccharis* species. (**a**–**h**). *Baccharis* species in nature. (**i**–**q**). cross-sections of *Baccharis* species, showing the axis and number of wings. (**a**,**i**). *B. articulata*; (**b**,**j**). *B. milleflora*; (**c**,**k**). *B. myriocephala*; (**d**,**l**–**n**). *B. pentaptera*; (**e**,**o**). *B. riograndensis*; (**f**,**p**). *B. sagittalis*; (**g**,**h**,**q**). *B. trimera*. [ax: axis, pi: pith, wi: wing]. Scale bar: (**a**,**h**): 1 cm, (**b**–**g**): 5 cm, (**i**–**q**): 500 µm.

The morpho-anatomical features of the cladodes from seven species of *Baccharis* were compared and the main characteristics that support the differentiation are summarized in Table 2. The morphological features were similar among the species (Figure 1a–h). Difficulty arises due to their shared possession of cladodes, which facilitates misidentification/adulteration.

All the analyzed species possess cladodes, although the number of wings can vary. *Baccharis articulata* (Figure 1a) features two wings (Figure 1a,i) measuring 0.3–0.6 cm in width, while *B. milleflora* (Figure 1b) has three wings (Figure 1b,j) and ranges from 0.5 to 2 cm in width. *B. myriocephala* (Figure 1c) presents three wings (Figure 1c,k) measuring 0.2–0.4 cm in width, whereas *B. pentaptera* (Figure 1d) possess two wings (Figure 1d,l) in the reproductive stems (0.2–0.4 cm width) and 3-(5)-wings (Figure 1m,n) in the vegetative stems. *Baccharis riograndensis* (Figure 1e) exhibits three winged stems (Figure 1e,o) measuring 0.2–0.5 cm wide, *B. sagittalis* (Figure 1f) has three wings (Figure 1f,p) (0.5–2 cm width), and *B. trimera* (Figure 1g,h) also possesses three wings (Figure 1g,h,q), measuring 0.3–1 cm in width.

Even though morphological differentiation is possible when flowering or fruiting, carquejas are commonly marketed in the form of dried fragmented stems, making the accurate identification of the species challenging. However, given the way carquejas are commercialized, microscopic characteristics become essential in their identification and quality control.

**Figure 2:**
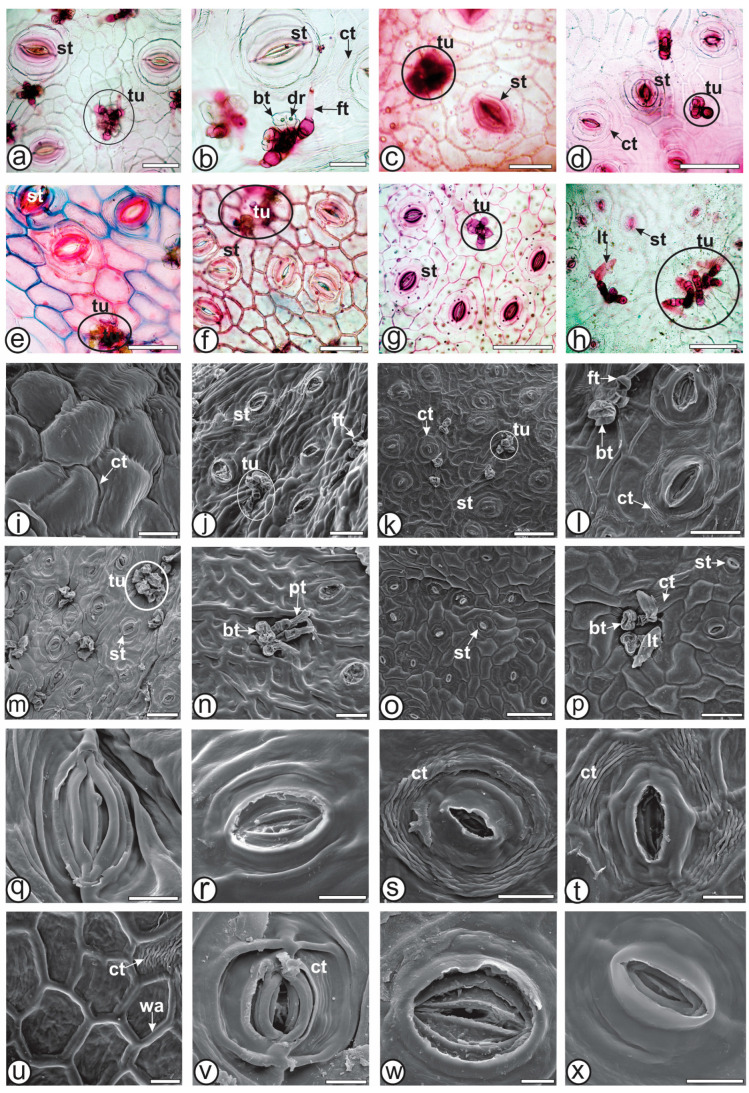
Anatomy of cladodes of *Baccharis* species—epidermis frontal view. (**a**–**h**). Light microscopy; stained in safranin. (**i**–**x**). SEM. (**a**,**b**,**i**,**q**). *B. articulata*; (**c**,**j**,**r**). *B. milleflora*; (**d**,**k**,**s**). *B. myriocephala*; (**e**,**l**,**t**,**u**). *B. pentaptera*; (**f**,**m**,**v**). *B. riograndensis*; (**g**,**n**,**w**). *B. sagittalis*; (**h**,**o**,**p**,**x**). *B. trimera*. [bt; biseriate glandular trichome, ct: cuticle, dr: druses, ft: flagelliform glandular trichome, lt: clavate non-glandular trichome, pt: spatulate glandular trichome, st: stomata, tu: trichomes in tufts, wa: cell wall]. Scale bars: (**a**,**c**–**h**,**j**,**l**–**n**,**p**): 50 µm; (**b**): 25 µm; (**i**,**q**–**u**): 20 µm; (**k**,**o**): 100 µm; (**v**–**x**): 10 µm.

Epidermal characteristics, including the cuticle, epidermal cell walls, stomata, and trichomes have been recognized as significant tools in delineating the *Baccharis* genus [4,5,13]. Only *B. sagittalis* (Figure 2g,o) and *B. trimera* (Figure 2p) show wavy anticlinal epidermal cell walls, whereas the remaining species present straight to slightly wavy anticlinal epidermal cell walls (Figure 2a–f,i–n). Also, the anticlinal epidermal cell wall is conspicuously raised above the cladode surfaces in *B. myriocephala* (Figure 2s) and *B. pentaptera* (Figure 2l,t,u). This characteristic is also found in *B. riograndensis*, yet only around the stomata (Figure 2m,v), differentiating it from other carquejas.

*Baccharis milleflora* (Figure 2j), *B. sagittalis* (Figure 2o) and *B. trimera* (Figure 2p) possess smooth or slightly striated cuticles. However, *B. articulata* (Figure 2i) showed striated cuticles, whereas *B. myriocephala*, *B. pentaptera* and *B. riograndensis* presented striations, especially as concentric rings around stomata (Figure 2k,l,m,s,t,v). This feature was also observed in *B. microdonta* and *B. sphenophylla* [5]. In the present study, cuticle striations were also found in a perpendicular direction around trichome bases in *B. myriocephala* (Figure 2k), *B. pentaptera* (Figure 2l), *B. riograndensis* (Figure 2m), *B. sagittalis*, and *B. trimera* (Figure 2p), as observed in *B. punctulata* [4]. Cuticle ornamentation can aid in differentiation when analyzed alongside other features.

The type of stomata can help species identification in *Baccharis* [4], as also observed in this study. Freire et al. [13] found the following six types of stomata: anomocytic, anisocytic, cyclocytic, actinocytic, tetracytic, and staurocytic in *Baccharis*. In the present study, stomata (Figure 2a–h,j–t,v–x) of the anomocytic type were found in all studied species, paratetracytic stomata were found in *B. milleflora* (Figure 2c), cyclocytic in *B. articulata* (Figure 2a,b), *B. milleflora*, *B. myriocephala* (Figure 2d) and *B. sagittalis* (Figure 2g). Anisocytic stomata were present in *B. pentaptera*, *B. riograndensis* and *B. trimera* (Figure 2o,p), tetracytic in *B. articulata* and *B. myriocephala* and paracytic were found in *B. sagittalis*.

Rodriguez, Gattuso, and Gattuso [18] affirmed that stomatal size and stomatal index can help differentiate various *Baccharis* species. These features display substantial variation among the examined *Baccharis* species and can provide key anatomical distinction. *Baccharis trimera* demonstrates the highest stomatal index (15.2 ± 0.91d), statistically distinct from all other species, followed by *B. riograndensis* (10.6 ± 0.96c) and *B. pentaptera* (9.1 ± 0.87c). Otherwise, *B. milleflora* has the lowest stomatal index (3.5 ± 0.70b) compared to the other species. The intermediate values observed in *B. articulata* (5.3 ± 0.67a), *B. myriocephala* (5.9 ± 0.73a), and *B. sagittalis* (5.7 ± 0.94a) suggest some overlapping in this anatomical parameter. Stomatal size, measured as the average length and width, further differentiates these species. *B. articulata* and *B. milleflora* exhibit the largest stomata, with dimensions of 51.8 ± 1.95a × 32.5 ± 1.51A and 48.0 ± 2.09a × 27.8 ± 2.71B, respectively, both statistically larger than those of other species. Conversely, *B. trimera* shows the smallest stomata (10.6 ± 1.14e × 7.30 ± 0.58E), significantly different from the others, indicating a marked reduction in both length and width. Species like *B. myriocephala*, *B. pentaptera*, and *B. riograndensis* show intermediate stomatal sizes, but statistically significant differences, as indicated by the corresponding letters. These results suggest that stomatal size, alongside the stomatal index, contributes to the functional differentiation among species (Table 2).

Freire et al. [13] studied 38 *Baccharis* species, identified 7 types of trichomes, and stated that they are conical, aseptate flagellate, simple, branched, filiform flagellate, 1-armed and 2–4-armed. These authors affirmed that trichomes are regarded as the most significant anatomical markers for identifying the *Baccharis* species, while other characteristics, such as stomata type and epidermal cell-wall structure, can assist in differentiation as secondary features. Trichomes grouped in tufts are unique within the Asteraceae family [43]. In most species of the genus *Baccharis*, these tufts are uniformly distributed across the epidermis of the leaves and stems. Typically, these tufts comprise both uniseriate (frequently described as non-glandular) and glandular trichomes [4,5,44].

**Figure 3:**
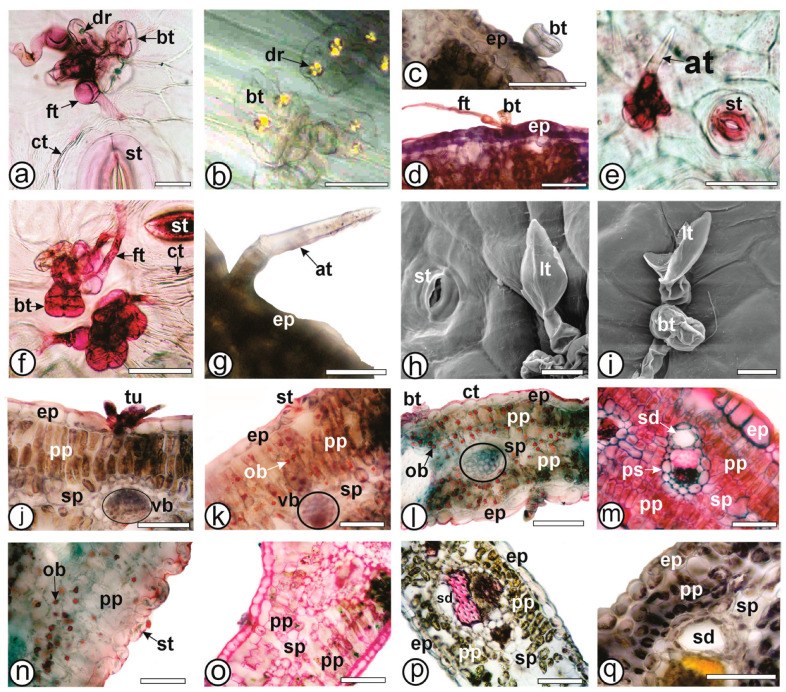
Anatomy of cladodes of *Baccharis* species. (**a**,**b**,**e**,**f**,**h**,**i**). Wing epidermis frontal view. (**c**,**d**,**g**,**j–q**). Wing cross-section. (**b**). Polarized light microscopy. (**a**,**c**–**g**,**j**–**q**). Light microscopy. (**h**,**i**). SEM. (**a**,**b**,**j**). *B. articulata*; (**c**,**d**,**k**). *B. milleflora*; (**e**,**l**). *B. myriocephala*; (**f**,**m**). *B. pentaptera*; (**g**,**n**). *B. riograndensis*; (**o**). *B. sagittalis*; (**h**,**i**,**p**,**q**). *B. trimera*. [at: armed non-glandular trichome, bt: biseriate glandular trichome, ct: cuticle, dr: druses, ep: epidermis, ft: flagelliform glandular trichome, lt: clavate non-glandular trichome, ob: oil bodies; pp: palisade parenchyma; sd: secretory duct; sp: spongy parenchyma, st: stomata, tu: trichomes in tufts, vb: vascular bundle]. Scale bars: (**a**,**b**,**g**): 25 µm; (**c**–**f**,**j**–**q**): 50 µm; (**h**,**i**): 20 µm.

In agreement with the literature, trichome tufts were found in the seven carquejas analyzed in this study (Figure 2a–h,j–n,p). Biseriate glandular trichomes are present in all species and are composed of two pairs of basal cells and a head containing up to four pairs of secretory cells (Figure 2b and Figure 3a–c). They can be found solitarily or in clusters with similar trichomes, forming tufts. Histochemical testing with Sudan III confirmed the presence of lipophilic content. A variation of biseriate glandular trichomes containing druse crystals within the secretory head cells were observed in *B. articulata* [45,46], *B. microdonta*, *B. punctulata*, and *B. sphenophylla* [4]. In the present study, this feature is observed only in *B. articulata* (Figure 2b and Figure 3b), and thus, it can be considered an anatomical marker for this species in relation to the other studied carquejas.

Four types of uniseriate trichomes are identified. The first type is a flagellate glandular trichome, frequently documented in *Baccharis* species [4,5,16]. The trichome body is secretory, voluminous, and composed of 4–6 rectangular cells, with a whip-like, tubular, translucent apical cell containing dense lipophilic substances. They are found in *B. articulata* (Figure 3a), *B. milleflora* (Figure 3d), and *B. pentaptera* (Figure 2l and Figure 3f). In *B. milleflora*, the apical cell is longer than in *B. articulata* and *B. pentaptera*.

The second type of uniseriate trichome is the armed non-glandular trichome, which consists of 2–3 cells in the body and has a sharply pointed apical cell with thick walls. This trichome type is found in *B. myriocephala* (Figure 3e) and *B. riograndensis* (Figure 3g). Armed trichomes have also been observed in other carquejas, such as *B. crispa* [18,19] and *B. triangularis* [22]. The third type of uniseriate glandular trichome is spatula-shaped, consisting of 3–4 body cells with thin cell walls and ending in a cylindrical, rounded tip cell that is as long as the body hair (basal cells). The apical cell is slightly narrower than the subterminal cell. This trichome type is present in *B. sagittalis* (Figure 2n) and has been documented in *B. penningtonii*, *B. sagittalis*, and *B. phyteumoides* [19]. Finally, the fourth type of uniseriate trichome is the clavate-shaped non-glandular trichome, composed of 3–4 body cells and an apical cell shaped like a club. It is present in *B. trimera* (Figure 2h,p and Figure 3h,i) and has also been observed by previous authors [16,19,22].

Focusing solely on the types of trichomes, biseriate glandular trichomes with a pair of druses in *Baccharis articulata*, uniseriate spatulate glandular trichomes in *B. sagittalis*, and uniseriate clavate non-glandular trichomes in *B. trimera* can be easily distinguished from the other carqueja species. However, for *B. milleflora*, *B. myriocephala*, *B. pentaptera*, and *B. riograndensis*, additional anatomical markers are needed to differentiate these species effectively.

The density of tufted trichomes differs significantly across the *Baccharis* species, as indicated by the statistical differences marked by different letters. *Baccharis pentaptera* showed the highest tufted trichome density (9.9 ± 0.73d), closely followed by *B. riograndensis* (9.1 ± 0.87d), with both species exhibiting significantly higher densities than the others. On the other hand, *B. milleflora* (2.00 ± 0.66b), *B. trimera* (2.1 ± 0.56b), and *B. sagittalis* (2.2 ± 0.63bc) presented the lowest densities, which are statistically similar. *B. articulata* (6.1 ± 0.99a) and *B. myriocephala* (3.3 ± 0.48c) depicted intermediate values.

The wings, in a cross-section, possess a single-layered epidermis covered by a thin cuticle (Figure 3j–q) that reacted with Sudan III in the histochemical tests (Figure 3l,n). Regarding the photosynthetic parenchyma, all the species show isobilateral organization (Figure 3j–q), comprising two layers of palisade on both sides and two layers of spongy parenchyma in the central region. This characteristic has been reported for several species of carquejas [5,16,22]. Oil bodies are present in chlorenchyma (palisade and spongy) of *B. milleflora* (Figure 3k), *B. myriocephala* (Figure 3l) and *B. riograndensis* (Figure 3n). This is the first report describing oil bodies in carqueja cladodes, which support species identification. However, Budel [4] reported oil bodies in the leaves of *B. illinita*, *B. microdonta*, *B. punctulata*, *B. reticularioides*, and *B. sphenophylla*.

Minor collateral vascular bundles surrounded by a parenchymatic sheath cross the spongy parenchyma (Figure 3j–m,p,q). Some minor vascular bundles are arranged in an inverted manner. One or more secretory ducts (Figure 3m,q) composed of a uniseriate epithelium, dense cytoplasm, evident nucleus, and lipophilic content can be found next to the parenchymatic sheath towards the phloem. Similar secretory ducts were commonly reported in the *Baccharis* species [16,22,44].

**Figure 4:**
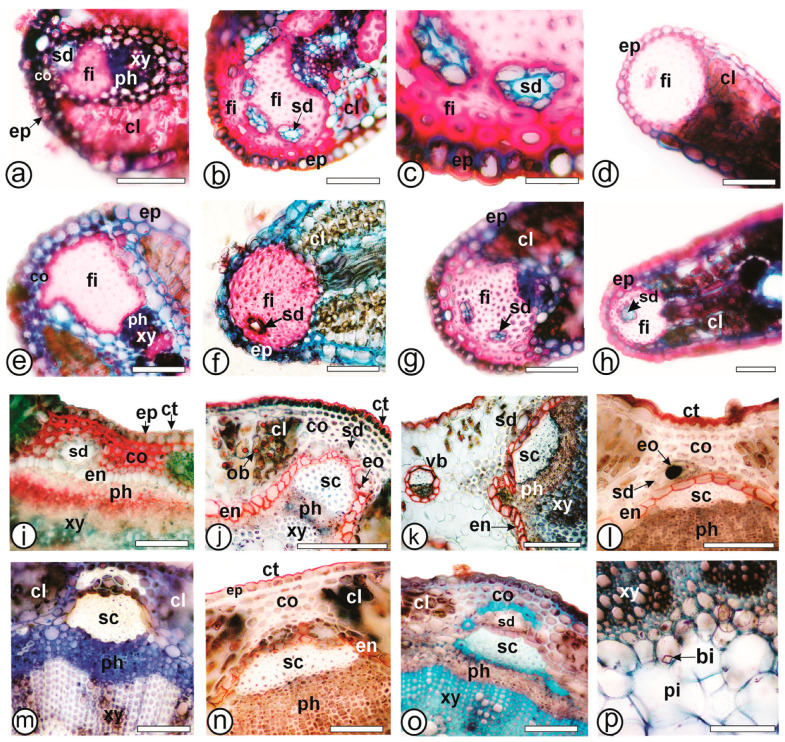
Anatomy of cladodes of *Baccharis* species—cross-section. (**a**–**h**). Wing edges. (**i**–**p**). Central axis. (**a,i**). *B. articulata*; (**b**,**c**,**j**). *B. milleflora*; (**d**,**k**). *B. myriocephala*; (**e**,**l**). *B. pentaptera*; (**f**,**m**). *B. riograndensis*; (**g**,**n**). *B. sagittalis*; (**h**,**o**,**p**). *B. trimera*. [bi: bipyramidal crystal, cl: chlorenchyma, co: collenchyma, ct: cuticle, en: endodermis, eo: essential oil, ep: epidermis, fi: fibers, ob: oil bodies, ph: phloem, pi: pith, sd: secretory duct, vb: vascular bundle, xy: xylem]. Scale bar: (**a**,**b**,**e**–**g**,**j**,**l**–**n**,**p**): 50 µm; (**c**): 10 µm; (**d**,**h**): 200 µm; (**i**): 20 µm (**o**,**k**): 100 µm.

At the wing edges, a single layer of angular collenchyma is present below the epidermis in *B. articulata* (Figure 4a) and *B. pentaptera* (Figure 4e), distinguishing them from other carquejas studied (Table 3). This characteristic was also observed in *B. microcephala* and *B. penningtonii* by Martínez [19], who noted that the presence or absence of subepidermal collenchyma in the wing edges is a distinguishing feature among certain carqueja species, as observed in the present study.

All species exhibit a collateral vascular bundle with a perivascular fiber cap adjacent to the phloem (Figure 4a–h), and secretory ducts are absent (Figure 4d,e) or present in varying numbers in the wing edges of *B. articulata* (Figure 4a), *B. milleflora* (Figure 4b,c), *B. riograndensis* (Figure 4f), *B. sagittalis* (Figure 4g), and *B. trimera* (Figure 4h). The number of secretory ducts appears to vary, as observed in *B. trimera* by Minteguiaga et al. [22]. For all species, the epidermis of the stem axis exhibits features like those of the wings. Below the epidermis, the chlorenchyma alternates with angular collenchyma, which consists of 2–5 rows in the ribs (Figure 4i–o). Secretory ducts, similar to those observed in the wings, are found adjacent to the endodermis, which bounds the internal part of the cortex and has walls impregnated with lipophilic compounds (Figure 4i–o). The vascular cylinder contains a cambium-producing xylem inward and outward phloem (Figure 4i–k,m–o). Perivascular fiber caps are adjacent to the phloem, and some fibers can also be found within the phloem. The pith is composed of relatively large, thin-walled parenchymatous cells (Figure 4p), containing crystals.

### 3.2. Histochemical Analysis

Ferric chloride and potassium dichromate react positively for phenolic compounds in all species of the present study, especially in the chlorenchyma cells. Sudan III, Sudan black and Oil red staining was visible on the lipophilic compounds in the cuticle, glandular trichomes, secretory epithelium of the ducts, and the endodermis of all species (Figure 4l,n), as well as the oil bodies in *B. milleflora* (Figure 3k), *B. myriocephala* (Figure 3l) and *B. riograndensis* (Figure 3n). Iodine solution evidenced starch grains in the chlorenchyma and collenchyma in *B. myriocephala* and *B. trimera*. Phloroglucinol/HCl reacted with lignified elements such as fibers and xylem vessels in all species (Figure 1k). Ruthenium red reacted with pectin and was detected especially in the collenchyma (Figure 3o and Figure 4i).

Coomassie Brilliant Blue and Xylidine Ponceau were used to detect protein bodies, which were not found in the analyzed species. The studied species did not react with vanillin/HCl for condensed tannins and with Dragendorff, Ellram, and Wagner for alkaloids. The Schiff reagent showed a positive reaction for polysaccharides in cell walls, collenchyma, and pith.

### 3.3. Crystal Analysis

**Figure 5:**
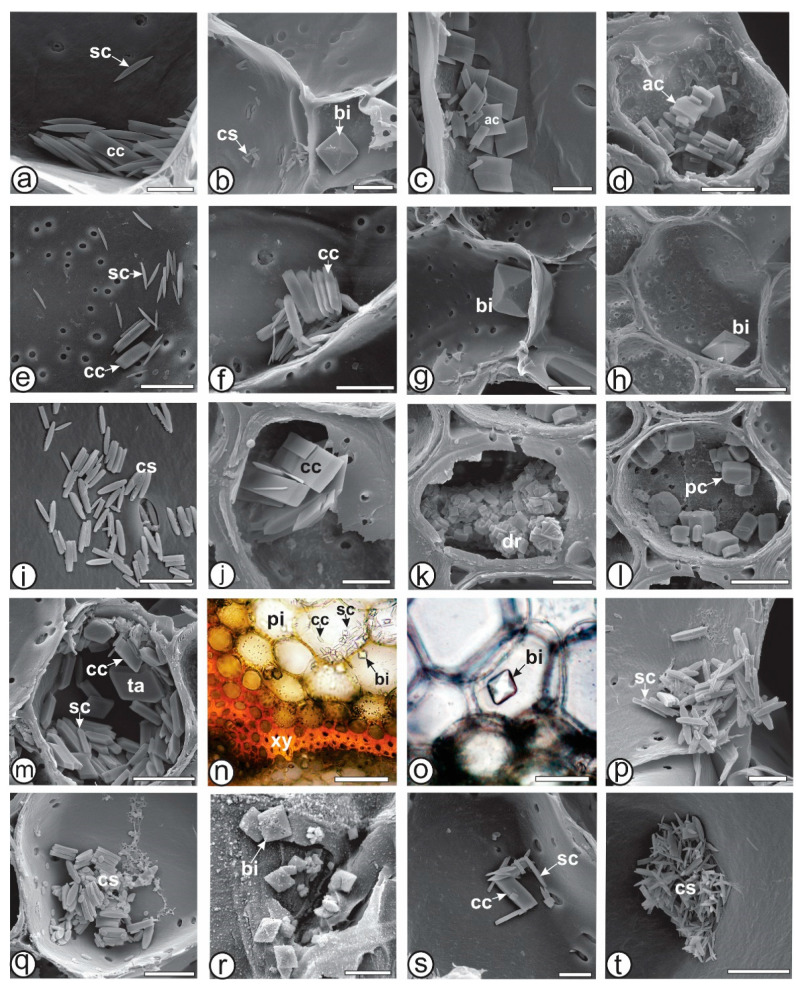
Crystals found in the cladodes of *Baccharis*, (**n**,**o**). Light microscopy, (**a**–**m**,**p**–**t**). SEM, (**a**–**d**). *B. articulata*, (**e**–**h**). *B. milleflora*, (**i**–**l**). *B. myriocephala*, (**m**–**o**). *B. pentaptera*, (**p**,**q**). *B. riograndensis*, (**r**). *B. sagittalis*, (**s**,**t**). *B. trimera*. [ac: arrow crystal; bi: bipyramidal; cc: cuneiform; cs: crystalline sand; dr: druses; pc: prismatic quadrangular; pi: pith; sc: styloid; ta: tabular; xy: xylem]. Scale bar: (**a**,**c**,**d**,**i**–**k**,**p**,**r**,**s**): 5 µm; (**b**,**e**–**g**,**l**,**m**,**q**,**t**): 10 µm; (**h**): 20 µm; (**n**): 50 µm; (**o**): 5 µm.

The shape and location of crystals within particular taxa are consistent and specific, allowing them to be used as anatomical markers for the identification of a species, a section, or even a genus [47,48]. Recently, Raeski et al. [46] studied forty-four species of *Baccharis* and provided a comprehensive account of the morphotypes of calcium oxalate crystals found within the genus. In a subsequent study, Raeski et al. [45] detailed eleven types of crystals in *B. articulata*, including bipyramidal (simple and rectangular), pyramidal rectangular, cuneiform, tabular, arrow-shaped, crystalline sands, styloids, and druses. They asserted that these crystal types can serve as anatomical markers for the carqueja complex within the *Baccharis* genus. In the present study, the same morphotypes were found in *B. articulata* and they were compared to the other carquejas, as summarized in Table 4. Arrow-shaped, bipyramidal rectangular and rhomboidal crystals were observed exclusively in *B. articulata*. Bipyramidal simple crystals were found in *B. articulata*, *B. milleflora*, *B. pentaptera* and *B. sagittalis*. Crystalline sand was found in *B. articulata*, *B. myriocephala*, *B. riograndensis* and *B. trimera*. Cuneiform crystals were found in *B. articulata*, *B. milleflora*, *B. myriocephala*, *B. pentaptera* and *B. trimera*. Druses were found in *B. articulata* and *B. myriocephala*, while prismatic quadrangular crystals were observed only in *B. myriocephala*. Styloids were found in *B. articulata*, *B. milleflora*, *B. pentaptera*, *B. riograndensis* and *B. trimera*. Tabular crystals were found in *B. articulata* and *B. pentaptera*. The shape of the crystals is shown in Figure 5.

**Figure 6:**
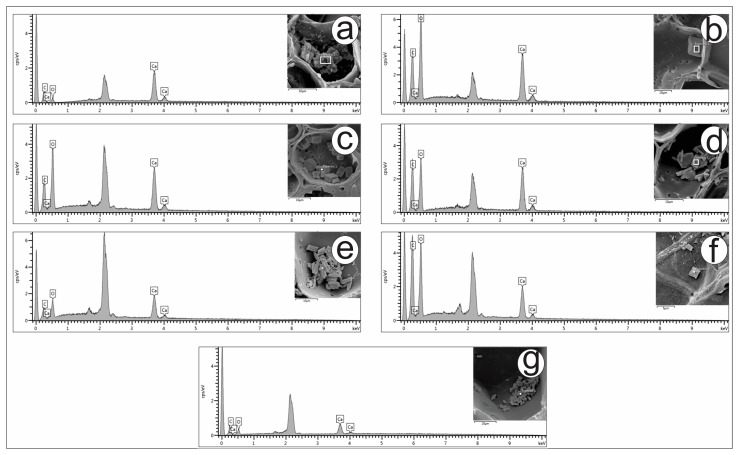
EDS spectra of crystals found in the cladodes of *Baccharis* species. *B. articulata* = (**a**); *B. milleflora* = (**b**); *B. myriocephala* = (**c**); *B. pentaptera* = (**d**); *B. riograndensis* = (**e**); *B. sagittalis* = (**f**); *B. trimera* = (**g**).

The elemental chemical composition of crystals in plants has been investigated by several researchers using EDS. Most studies reported the presence of calcium oxalate in those crystals [45,46,49]. Using EDS, it was possible to conclude that the composition of the crystals of the carqueja species is calcium oxalate, as shown in Figure 6.

The prominent unlabeled peak near 2 keV represents gold (Au), the metal used to sputter-coat the samples.

Several studies have demonstrated the importance and effectiveness of morpho-anatomy in identifying and differentiating species with similar morphology, even when the plants are fragmented [16,44,49]. In the present study, several anatomical parameters were identified as potential anatomical markers for the identification and differentiation of species, though these findings should be validated through analyses involving a larger number of populations. While some characteristics may be universally present within a taxon, others could arise from specific individuals responding to unaccounted environmental factors. Additionally, among those seven species of carqueja, only *B. articulata*, *B. milleflora*, and *B. trimera* have some studies proving their efficacy and safety to use as a digestive and a diuretic [8,50,51]. The other species—*B. pentaptera*, *B. myriocephala*, *B. sagittalis*, and *B. riograndensis*—lack such studies. The present study can help differentiate these species, reducing the risks of using those with no therapeutic efficacy and safety studies.

## 4. Conclusions

Among the seven species of carquejas analyzed, several features have potential to be used as markers for the delimitation of the species. Of these, the presence or absence of oil bodies, the subepidermal collenchyma layer at the wing edge, and the trichome types are the most significant and discriminatory characteristics. *Baccharis articulata* can be distinguished by the presence of biseriate glandular trichomes containing a pair of druses within the apical cells. *Baccharis milleflora* and *B. pentaptera* both have uniseriate flagelliform trichomes but can be differentiated by the presence of oil bodies in the chlorenchyma of the wings and stem axis in *B. milleflora*. *Baccharis myriocephala* and *B. riograndensis* possess uniseriate armed trichomes, yet they can be distinguished from each other by the presence of a collenchyma layer at the wing edges in *B. riograndensis*. Only *B. sagittalis* has uniseriate spatula-shaped trichomes, and only *B. trimera* exhibits uniseriate clavate-shaped trichomes. The stomatal index varies among the *Baccharis* species, indicating distinct anatomical data. *Baccharis trimera* demonstrates the highest stomatal index, while *B. milleflora* has the lowest.

These characteristics can be observed easily, even in the fragmented materials sold in the market. If the plants are commercialized in powder form, it is suggested that other techniques, such as HPTLC, may be used in combination with microscopy.

Given the large number of morphologically similar species in the complex, it is crucial to study other species of carquejas as well in the future, to ensure the quality control of raw material and herbal products. In addition, further investigations should be carried out with a larger number of populations and localities to confirm these anatomical markers. Comprehensive studies involving multiple samples obtained from different populations are needed to understand the variations within and between species influenced environmental and edaphic factors.

## Figures and Tables

**Table 1:** Collection details of species used in this study.

Species of *Baccharis*	Herbarium Code	Location
*B. articulata* (Lam.) Pers.	ECT0006351	Pelotas—RS
*B. milleflora* (Less.) DC.	ICN192141	Ponta Grossa—PR
*B. myriocephala* DC.	ECT0009951	Morretes—PR
*B. pentaptera* (Less.) DC.	ECT0009952	Piraquara—PR
*B. riograndensis* Malag. and J.Vidal	ECT0009954	Pelotas—RS
*B. sagittalis* (Less.) DC.	ECT0009953	Morretes—PR
*B. trimera* (Less.) DC.	ICN129479	Ponta Grossa—PR

**Table 2:** Anatomy features to differentiating between the *carqueja* species (*Baccharis*, Asteraceae).

Anatomy Features	*B. articulata*	*B. milleflora*	*B. myriocephala*	*B. pentaptera*	*B. riograndensis*	*B. sagittalis*	*B. trimera*
Number of wing/width in cm	2/0.3–0.6	3/0.5–2	3/0.2–0.4	2, 3 or 5/0.2–0.4	3/0.2–0.5	3/0.5–2	3/0.3–1
Epidermal anticlinal cell walls	straight to slightly wavy	straight to slightly wavy	straight to slightly wavy (raised above the cladode surfaces)		straight to slightly wavy (raised above the stomata)	Wavy	
Cuticle	striated	smooth to slightly striated	striations, especially as concentric rings around stomata and in a perpendicular direction around trichome bases			smooth to the slightly striated and in a perpendicular direction around trichome bases	
Stomata	Anomocytic, anisocytic, cyclocytic and tetracytic	Anomocytic, cyclocytic and paratetracytic	Anomocytic, anisocytic, cyclocytic and tetracytic	Anomocytic and anisocytic		Anomocytic, anisocytic, cyclocytic and paracytic	Anomocytic and anisocytic
Stomata index	5.3 ± 0.67 ^a^	3.5 ± 0.70 ^b^	5.9 ± 0.73 ^a^	9.1 ± 0.87 ^c^	10.6 ± 0.96 ^c^	5.7 ± 0.94 ^a^	15.2 ± 0.91 ^d^
Stomata size (average length × width in μm)	51.8 ± 1.95 ^a^ × 32.5 ± 1.51 ^A^	48.0 ± 2.09 ^a^ × 27.8 ± 2.71 ^B^	19.5 ± 1.58 ^b^ × 12.3 ± 2.00 ^C^	35.4 ± 1.51 ^c^ × 21.0 ± 3.46 ^D^	35.2 ± 1.78 ^c^ × 21.2 ± 2.28 ^D^	25.4 ± 0.93 ^d^ × 13.1 ± 1.30 ^C^	10.6 ± 1.14 ^e^ × 7.30 ± 0.58 ^E^

Note: Different letters indicate statistically significant difference at *p* < 0.05 (one-way ANOVA with post hoc Tukey’s HSD test); mean and standard deviation from 10 fields at 40× magnification.

**Table 3:** The anatomical features of trichomes and subepidermal collenchyma at the wing edge to differentiate *carqueja* species (*Baccharis*, Asteraceae).

Anatomy Features	*B. articulata*	*B. milleflora*	*B. myriocephala*	*B. pentaptera*	*B. riograndensis*	*B. sagittalis*	*B. trimera*
Biseriate glandular trichome with a pair of druses	+	−	−	−	−	−	−
Uniseriate flagelliform glandular trichome	+	+	−	+	−	−	−
Uniseriate armed non-glandular trichome	−	−	+	−	+	−	−
Uniseriate spatulate glandular trichome	−	−	−	−	−	+	−
Uniseriate clavate non-glandular trichome	−	−	−	−	−	−	+
Tufted trichome density	6.1 ± 0.99 ^a^	2.00 ± 0.66 ^b^	3.3 ± 0.48 ^c^	9.9 ± 0.73 ^d^	9.1 ± 0.87 ^d^	2.2 ± 0.63 ^b,c^	2.1 ± 0.56 ^b^
Oil bodies in chlorenchyma	−	+	+	−	+	−	−
Angular collenchyma in the wing edges	+	−	−	+	−	−	−

Note: + present; − absent; different letters indicate statistically significant differences at *p* < 0.05 (one-way ANOVA with post hoc Tukey’s HSD test); mean and standard deviation from 10 fields at 40× magnification.

**Table 4:** Crystal types found in *carqueja* species.

Crystal Type	*B. articulata*	*B. milleflora*	*B. myriocephala*	*B. pentaptera*	*B. riograndensis*	*B. sagittalis*	*B. trimera*
Arrow-shaped	+	−	−	−	−	−	−
Bipyramidal rectangular	+	−	−	−	−	−	−
Bipyramidal simple	+	+	−	+	−	+	−
Crystalline sand	+	−	+	−	+	−	+
Cuneiform	+	+	+	+	−	−	+
Druse	+	−	+	−	−	−	−
Prismatic quadrangular	−	−	+	−	−	−	−
Rhomboidal	+	−	−	−	−	−	−
Styloid	+	+	−	+	+	−	+
Tabular	+	−	−	+	−	−	−

+ Present; − Absent.

## Data Availability

The data are contained within the article.
